# Efficacy and Safety of Ketogenic Diet Treatment in Pediatric Patients with Mitochondrial Disease

**DOI:** 10.3390/nu16060812

**Published:** 2024-03-13

**Authors:** Dorota Wesół-Kucharska, Milena Greczan, Magdalena Kaczor, Ewa Ehmke vel Emczyńska-Seliga, Małgorzata Hajdacka, Edyta Czekuć-Kryśkiewicz, Dorota Piekutowska-Abramczuk, Paulina Halat-Wolska, Elżbieta Ciara, Maciej Jaworski, Aleksandra Jezela-Stanek, Dariusz Rokicki

**Affiliations:** 1 Department of Pediatrics, Nutrition and Metabolic Diseases, The Children’s Memorial Health Institute, Al. Dzieci Polskich 20, 04-730 Warsaw, Poland; m.greczan@ipczd.pl (M.G.); m.kaczor@ipczd.pl (M.K.); e.emczynska@ipczd.pl (E.E.v.E.-S.); m.hajdacka@ipczd.pl (M.H.); d.rokicki@ipczd.pl (D.R.); 2 Laboratory of Radioimmunology and Experimental Medicine, Department of Biochemistry, Radioimmunology and Experimental Medicine, The Children’s Memorial Health Institute, Al. Dzieci Polskich 20, 04-730 Warsaw, Poland; e.czekuc-kryskiewicz@ipczd.pl; 3 Department of Medical Genetics, The Children’s Memorial Health Institute, Al. Dzieci Polskich 20, 04-730 Warsaw, Poland; d.abramczuk@ipczd.pl (D.P.-A.); p.halat@ipczd.pl (P.H.-W.); e.ciara@ipczd.pl (E.C.); 4 Laboratory of Densitometry, Department of Biochemistry, Radioimmunology and Experimental Medicine, The Children’s Memorial Health Institute, Al. Dzieci Polskich 20, 04-730 Warsaw, Poland; m.jaworski@ipczd.pl; 5 Department of Genetics and Clinical Immunology, National Institute of Tuberculosis and Lung Diseases, 26 Plocka Str, 01-138 Warsaw, Poland; jezela@gmail.com

**Keywords:** mitochondrial diseases, ketogenic diet, IPMDS, nDNA, mtDNA

## Abstract

Mitochondrial diseases (MDs) are a heterogeneous group of disorders resulting from abnormal mitochondrial function. Currently, there is no causal treatment for MDs. The aim of the study was to assess the effectiveness and safety of the ketogenic diet (KD) in patients with MD and to analyse selected biochemical and clinical parameters evaluating the effectiveness of KD treatment in patients with MDs. A total of 42 paediatric patients were assigned to four groups: group 1—patients with MD in whom KD treatment was started (*n* = 11); group 2—patients with MD remaining on an ordinary diet (*n* = 10); group 3—patients without MD in whom KD treatment was initiated (*n* = 10), group 4—patients without MD on a regular diet (*n* = 11). Clinical improvement was observed in 9/11 patients with MD treated with KD. Among patients with MD without KD, the clinical condition deteriorated in 7/10 patients, improved in 2/10 patients, and remained unchanged in one patient. Adverse events of KD occurred with a comparable frequency in groups 1 and 3. There was no significant difference in changes in biomarker concentrations over the course of the study among patients treated and untreated with KD.

## 1. Introduction

Mitochondrial diseases (MDs) are a heterogeneous group of diseases resulting from impairment of mitochondrial structure or function, which leads to dysfunction of the respiratory chain and deficit in the production of energy in the form of ATP in the process of oxidative phosphorylation (OXPHOS) [1,2].

Both clinical and genetic heterogeneity characterise MDs—i.e., pathogenic variants in one gene may correspond to various diseases, but also one disease entity may result from the occurrence of molecular defects in different genes, both in nuclear DNA (nDNA) and mitochondrial DNA (mtDNA). The course of MD is usually progressive, with periods of exacerbations and remissions [1,3]. A characteristic feature is the worsening of symptoms at the time of infection, after physical effort, or during various stressful situations. The disease may be manifested at any age—from early childhood to old age. In more than a quarter of patients, the onset of MDs is in the neonatal period and infancy, and earlier disease manifestation is associated with a worse prognosis [4,5]. Dominant signs and symptoms in children are those resulting from central nervous system (CNS) damage, e.g., psychomotor development delay or regress, disorders in muscle tone, ataxia, myoclonus or seizures. Children with MD also typically show feeding disorders, body weight and height deficits, or recurrent vomiting [5,6].

### 1.1. Diagnosis of Mitochondrial Diseases

Diagnosis of mitochondrial diseases is complex and requires an in-depth analysis of clinical, biochemical, imaging and molecular data. No sufficiently sensitive and specific biochemical marker could be used to exclude or confirm MDs. Biochemical tests to be performed in diagnosing this group of diseases include serum and cerebrospinal fluid (CSF) levels of lactic acid (LA), pyruvic acid (PA) and alanine (ALA), organic acid excretion in urine using gas chromatography combined with mass spectrometry (GC/MS), or acylcarnitine profile from dried blood spot using tandem mass spectrometry (MSMS) [3,7,8,9]. Additional biomarkers whose sensitivity and specificity in diagnosing MDs were confirmed by many studies are fibroblast growth factor 21 (FGF21) and growth differentiation factor 15 (GDF15) [10,11]. FGF21 may also be used as a marker of the MDs stage; higher and gradually increasing concentrations were observed in subjects in the final stage of the disease and with more significant damage to skeletal muscles on histochemical examination (FGF21 level was correlated with a higher percentage of COX-negative fibres) [12,13,14]. It is suggested that FGF21 be used for monitoring the course and treatment of MDs [15,16].

### 1.2. Use of Ketogenic Diet in Patients with Mitochondrial Disease

A ketogenic diet involves the replacement of carbohydrates with lipids, which leads to the formation of ketone bodies [17,18], which represent an alternative source of energy for most cells of the body. A precise mechanism of KD has yet to be explained. It is known to improve cell metabolism by increasing energy production in the mitochondrial respiratory chain, restricting glycolysis, and increasing the oxidation of fatty acids in the cell. Additionally, it reduces the oxidation of coenzyme Q10, thereby reducing the amount of free radicals and oxidative stress. By increasing energy production in the cell, KD also increases hyperpolarisation of the cell membrane, suppresses glutamine release and affects neurotransmitters and ion channels in the CNS—this could explain, e.g., its antiepileptic activity [19,20,21]. KD is a reliable and recommended treatment method for drug-resistant epilepsy, both in adults and children, including infants [17,18,22]. Numerous publications point to a beneficial effect of KD in patients with epilepsy and MDs, especially in those patients where MDs result from damage to complex I of the respiratory chain [21,23]. Most studies, however, are based on a small group of patients, and often these are only case reports.

A ketogenic diet is not free of adverse events. Early KD complications include dehydration, gastrointestinal disorders (nausea, vomiting, diarrhoea, constipation), symptomatic hypoglycaemia, hyperuricemia and lipid disorders. Late complications (occurring more than four months after introducing KD) include osteopenia, nephrolithiasis, reduced carnitine level and iron deficiency anaemia. Most adverse reactions of KD are mild and short-term and regress spontaneously or require a minor dietary or pharmacological intervention [18,24]. Serious complications which require discontinuation of treatment with KD include acute pancreatitis, cardiomyopathy, long QT syndrome, protein-losing enteropathy and lipoid pneumonia [24,25].

The aim of the study was to assess the effectiveness of the ketogenic diet in paediatric patients with mitochondrial disease and to analyse selected biochemical and clinical parameters assessing the effectiveness of KD treatment in MD patients.

## 2. Materials and Methods

The study group comprised patients hospitalised at the Department of Paediatrics, Nutrition and Metabolic Diseases (DPNMD) CMHI in Warsaw aged between 1 month and 18 years. Patients were recruited into four groups:

Group 1: Patients with mitochondrial disease treated with a ketogenic diet.

Group 2: Patients with mitochondrial disease being on a regular diet.

Group 3: Patients without mitochondrial disease treated with ketogenic diet for other indications.

Group 4: Patients without mitochondrial disease being on a regular diet, without a ketogenic diet.

For patients with MD, the allocation to groups with or without KD (group 1 or group 2) was random. An exception was patients with confirmed or probable pyruvate dehydrogenase deficiency (PDHD), where KD is the treatment of choice—those patients were included in group 1 without randomisation. MD diagnosis was made based on clinical and biochemical data, including the Nijmegen score (a score of over four points is considered a probable disease) [7]. A definite diagnosis was such that it was confirmed in a molecular test. in patients in whom mitochondrial disease was not suspected (no elevated MD biomarkers, no typical changes for MD in neuroimaging, Nijmegen score below four) genetic tests were not performed—these patients were classified into group 4 or group 3 if there were medical indications for KD treatment (GLUT1 deficit or drug-resistant epilepsy).

To identify a pathogenic variant, confirming MD, selected molecular tests were performed in patients. Depending on clinical preconditions, the genetic analysis included: MLPA analysis and/or Sanger sequencing of selected mtDNA fragments or sequencing of selected genes for the identification of known MD depending on the presence of nDNA changes using an authorial “1000 genes” panel (next generation sequencing).

FGF21 was analysed using BioVendor Human FGF-21 ELISA assay (BioVendor—Laboratorní Medicína a.s., Brno, Czech Republic).

Patients with obesity (BMI > 97 percentile for age and sex), hepatic steatosis, cardiovascular diseases, patients treated with growth hormone, suffering from nephropathy, or patients in a critical condition (which results in FGF21 elevation) were not included in the study.

### 2.1. Course of Study

The study was prospective. Clinical evaluation and laboratory tests were performed at the time of qualification for the study (visit V0) and after 12 months (±1 month) (visit V12). At the time of inclusion in the study, all patients had the level of the following determined: FGF21, LA, PA, ALA, and creatine kinase (CK).

Patients with MD were assessed for MD severity using the International Paediatric Mitochondrial Disease Scale (IPMDS) [26]. The assessment was performed in three domains, then the score was summed up, and the total value (IMPDS total) was calculated. The higher the score (given as a percentage of the maximum score) on the presented scale, the more complaints and clinical symptoms occur in the patient, which means that MD is more severe.

**Figure 1:**
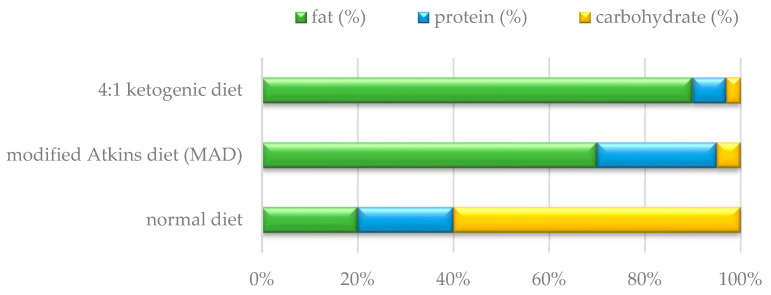
Nutrient ratios in the ketogenic diet, the modified Atkins diet and the normal diet.

KD was introduced in patients of groups 1 and 3 at V0. Before the inclusion of KD, caregivers received detailed information on the principles of such treatment. KD was included only if the caregivers and the patient accepted this method of nutrition and agreed to regular clinical and biochemical check-ups. KD was always introduced in hospital conditions. The clinical dietitian calculated the patient’s daily energy requirement, taking into account his age, gender and ideal body weight in accordance with the recommended dietary allowances (RDA). KD was started with a 1:1 ketogenic ratio (i.e., for 1 g of fat in the 24 h supply there was 1 g of protein and carbohydrates in total). The protein supply in the diet was appropriate to age—in accordance with RDA standards, the supply of fluids was not limited. No fasting was used. Meals were divided into 4–5 portions during the day. The meals took into account the patient’s dietary preferences. In the following days, the ketogenic ratio was gradually increased to 1.5:1 (1.5 g fat: 1 g no fat), up to a maximum of 4:1 (Figure 1). The ketogenic ratio in the diet was determined individually by controlling the level of β-hydroxybutyric acid (BHB) to achieve its therapeutic level (2–5 mmol/L). Blood gases (assessing for metabolic acidosis) and serum glucose levels (hypoglycaemia) were monitored daily. Before discharge, other biochemical parameters were assessed, including ALT, ASPT, urea, creatinine, total cholesterol, triglycerides, ionogram and blood count.

Additionally, the dietitian trained caregivers/parents in preparing meals with an appropriate ketogenic ratio at home from “ordinary” nutritional products and using ready-made ketogenic meals. KD treatment patients were invited for clinical and biochemical follow-up 4–6 weeks after discharge, and then every 3 months. Each time the patient’s menu was checked, clinical assessment was made, nutritional tolerance and the occurrence of adverse effects of the diet. Based on the 3-day menu, the dietitian calculated the actual ketogenic ratio of meals and, if necessary, the menu was modified to maintain the optimal concentration of ketones. After completing the study, caregivers had the option of continuing KD treatment or returning to their normal diet.

Patients without KD (groups 2 and 3) remained on their usual diet, with dietary standards (RDAs) adjusted for age, sex and body weight. If there were medical indications to do so, they also received dietary advice.

If the patient needed to discontinue treatment with KD (patient resignation from further treatment, occurrence of adverse effects of KD), the last visit on KD was considered V12.

No modification of additional treatment was made for MD patients, and the supply of previously taken dietary supplements was continued (e.g., coenzyme Q10, L-carnitine, thiamine and other vitamins). However, antiepileptic treatment was modified during the study, depending on the patient’s clinical condition, in accordance with the principles of treating epilepsy.

### 2.2. Statistical Methods

The chi-square test of independence with Yates correction (for group < 10) was used to check correlations between various clinical features in the study groups. To check statistical differences between parameters in the two groups, a comparison of 2 groups was made: Student’s test with Welch correction for unequal variances (test F for comparing variances in 2 groups); when comparing four groups, one-way analysis of variance was used. To assess biochemical parameters in particular groups, Kruskal–Wallis rank ANOVA was used with post hoc tests. Quantitative variables correlations were assessed using Spearman’s correlation test (they did not show normal distribution). The value of *p* < 0.05 was considered statistically significant.

The parent/legal guardian of the patients participating in the study gave their written consent to participate in the study. The study protocol and the informed consent forms for patients and their parents/statutory representatives were approved by the Bioethical Committee at CMHI (No. 40/KBE/2019 of 14 October 2019).

The study was funded by CMHI research grant no. 623/19.

## 3. Results

Sixty-three patients hospitalised at DPNMD CMHI in Warsaw were qualified for the study. The recruitment lasted 28 months (from October 2019 to the end of January 2022). The assumed patient follow-up period was 12 months. Twenty-one recruited children did not complete the study: fifteen subjects did not come for a follow-up visit (the study was conducted during the COVID-19 pandemic, which was a restriction or difficult for some patients to return for the scheduled follow-up visits), and six children died in the follow-up period of the mitochondrial disease. Table 1 presents the number of qualified subjects and those who completed the study in particular groups.

The study was completed by 42 children in total. Further analysis includes only these patients. The median age of the study subjects at the time of inclusion was 51 months (2–202 months), and the median follow-up period was 12 months (Table 1).

Group 1 (*n* = 11) comprised patients with MD who were randomised for the treatment with KD. In this group, six subjects had a molecularly confirmed pyruvate dehydrogenase deficiency (PDHD)—they were included in KD treatment without randomisation. Major clinical symptoms, age at inclusion in the treatment, follow-up period and genetic background of the disease are presented collectively for all patients in Supplement Table S1.

For all patients in group 1, treatment with KD was introduced at V0. Three children discontinued therapy due to adverse reactions of KD: acute pancreatitis (patient 1.1, after 13 months of treatment), hepatic steatosis and non-acceptance of the diet (patient 1.7, after five months of treatment) and due to persistent vomiting (patient 1.8, after eight months of treatment). In addition, patient 1.7 did not gain any benefit from the applied treatment. KD was well tolerated by the other subjects. For patient 1.9, there are only data up to month 5 of treatment; the child’s parents decided to continue treatment and care at a site closer to their living place.

The KD ratio in group 1 was 1.5:1 to 3.5:1; one patient had a modified Atkins diet (MAD). Median BHB level at V12 was: 2.50 mmol/L (range 1.23–4.0 mmol/L). For one patient, serum ketones were below the therapeutic value—1.23 mmol/L; the others achieved therapeutic values.

Group 2—comprised patients with MD who continued previous treatment. One patient (2.4) had a pathogenic variant in *PDHA1*; therefore, (after molecular findings) she was qualified for treatment with KD. In one patient, MD could not be confirmed by a genetic test, including the analyses mentioned above (patient 2.8); he received a Nijmegen score of 8, which suggests a very probable MD diagnosis [7,27].

Group 3 comprised patients for whom treatment with KD was introduced at V0 for reasons other than MD: drug-resistant epilepsy (*n* = 8) or deficiency of glucose transporter GLUT1 (pathogenic variant in gene *SLC2A1*) (*n* = 2). It was not possible to confirm clinical diagnosis by a molecular test for all the patients. In this group, two patients (3.4 and 3.9) discontinued treatment with KD after three months of therapy due to a lack of effects. The KD ratio ranged from 1.8:1 to 4:1; one patient received MAD. The median BHB level measured at V12 was: 3.81 mmol/L (0.1–4.9 mmol/L). In two patients, a therapeutic level of serum ketones was not achieved.

Group 4 in the presented study comprised patients diagnosed and/or treated at DPNMD for other reasons than MD. These patients had MDs ruled out on the basis of biomarker assessment, neuroimaging studies and a Nijmegen score of less than four. An additional genetic diagnosis was performed in some of them, which did not confirm the presence of a known pathogenic variant responsible for MDs (Table S1).

### 3.1. Efficacy of the Ketogenic Diet in Particular Groups

During the introduction of KD in patients of group 1 (MD patients), five patients were diagnosed with and treated for epilepsy, but only two patients had epileptic seizures (1.1 and 1.9). During the treatment with KD, in one patient, complete regression of the seizures was achieved, and in the other patient, a significant reduction (by 50–90%) was observed. In addition, in two of five patients receiving antiepileptic treatment, it was possible to withdraw antiepileptic drugs. In group 3 (KD without MD), a reduction in the number of epileptic seizures was achieved in six of eight patients with seizures during the introduction of KD, where one patient showed a complete decrease in seizures (3.8), four patients showed a reduction by 50–90%, and one patient by <50%. It was not possible to reduce the number of antiepileptic drugs or to withdraw them from anyone in this group. A statistically significant difference was observed with regard to muscle tone improvement, which was more often observed in group 1 than in group 3 (*p* < 0.05). A reduction in extra movements (trembling, ataxia or dystonic movements) was more often observed in patients treated with KD with MD than without MD, but the difference was not statistically significant (*p* = 0.5402) (Table 2).

### 3.2. Adverse Effects of the Ketogenic Diet

**Figure 2:**
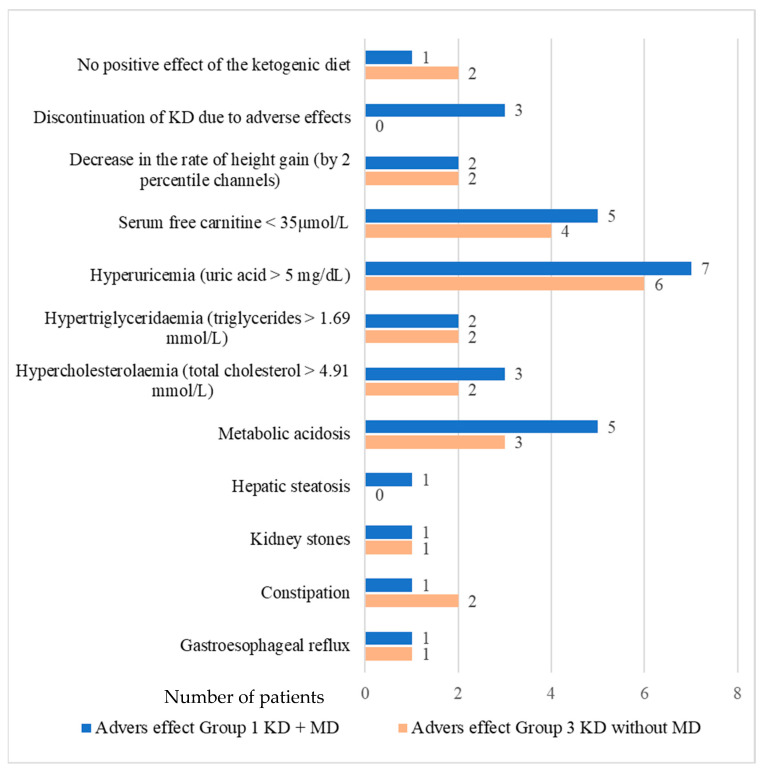
Adverse effects occurring during treatment with the ketogenic diet.

Adverse effects related to treatment with KD occurred with a comparable frequency in both treated groups and did not show a statistically significant difference. The most often reported were metabolic acidosis (on venous blood gas: pH < 7.350, HCO_3_ < 18 mmol/L and/or SB < −5), hyperuricaemia (>5 mg/dL) and reduced free carnitine level in serum (average: 35–75 µmol/L). Hypercholesterolemia (total cholesterol > 190 mg/dL (> 4.91 mmol/L) and hypertriglyceridaemia (triglycerides > 150 mg/dL, (>1.69 mmol/L) were mild—the maximum concentration of total cholesterol was 249 mg/dL (6.44 mmol/L). The maximum triglyceride concentration during the treated patients’ follow-up was 249 mg/dL (2.81 mmol/L). No patient had cardiac complications (e.g., cardiomyopathy or long QT syndrome). In none of the treated patients, neutropenia or symptomatic hypoglycaemia was revealed. A reduced weight gain was not observed, either, but a reduced height gain (height reduction by 2 percentile channels or <3 percentile for age and sex). In three patients with MD, KD was discontinued due to adverse effects. A lack of positive effect of the diet was found in one patient with MD (patient 1.7) and two patients without MD (patients 3.3 and 3.4). The observed adverse effects related to treatment with KD are shown in Figure 2.

### 3.3. Dynamics of Clinical Symptoms Occurring in Patients with Mitochondrial Disease Treated and Non-Treated with a Ketogenic Diet

**Figure 3:**
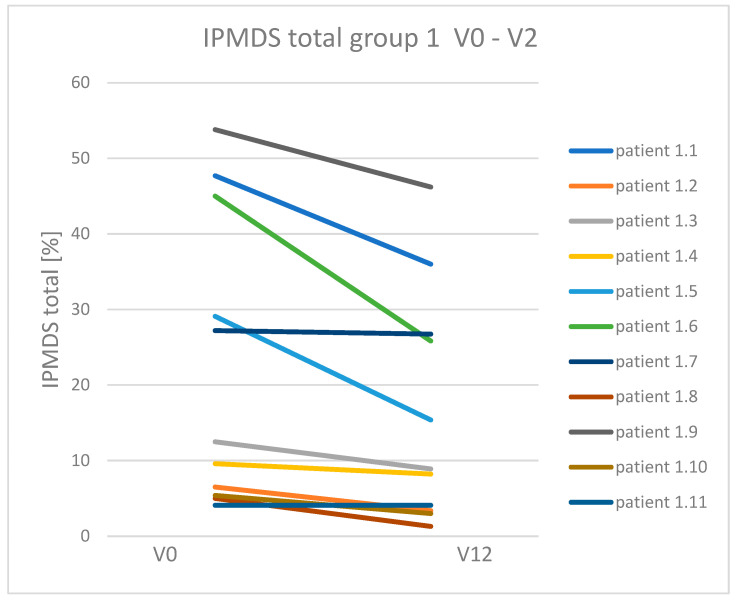

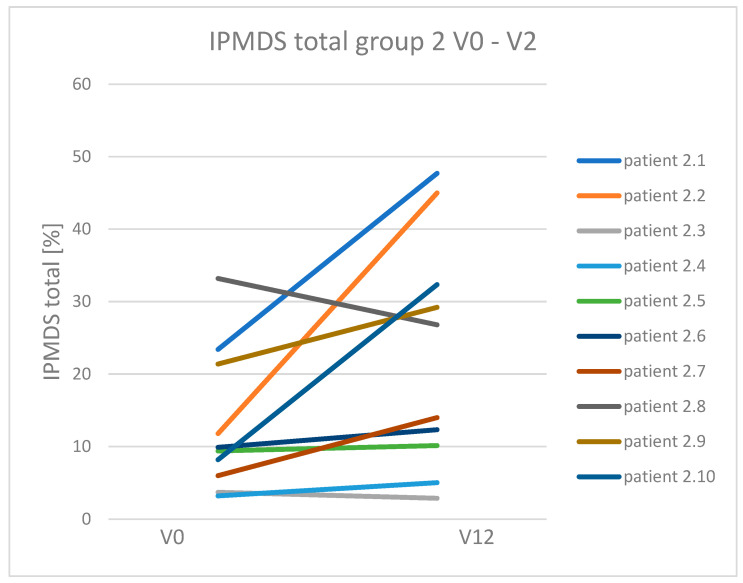
Change in IPMDS total score in patients in groups 1 and 2 at visits V0 and V12.

**Figure 4:**
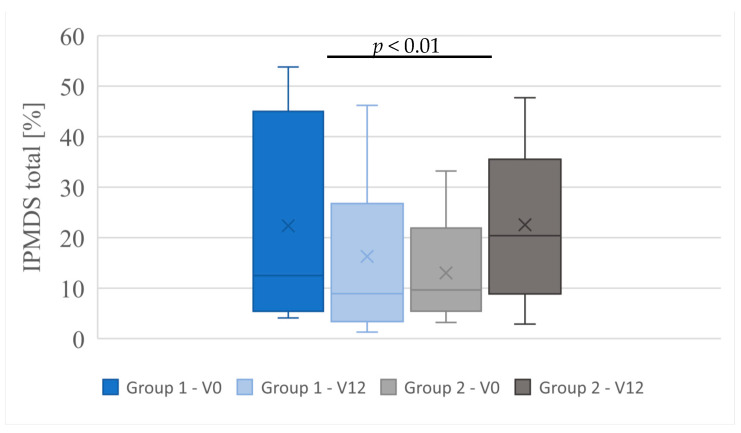
IPMDS total score in individual groups at visits V0 and V12, (test Kruskal-Wallis rank ANOVA with posthoc tests).

Clinical symptoms in MD patients treated and non-treated with KD were compared (group 1 vs. group 2). Group 1 with patients treated with KD, showed clinical improvement and reduction in symptoms compared to group 2. In nine patients receiving KD, the number of points on IPMDS was reduced (which means clinical improvement). In the other two patients in this group, the number of points did not change (their clinical condition was stable during the follow-up period). On the other hand, in group 2 (patients with MD non-treated with KD): two patients showed improvement; in one patient the condition was unchanged, and the other seven patients showed an increased number of points on IPMDS (which means worsening of clinical condition). Median points of IPMDS total in group 1 at V0 was 12.5% (4.1–53.8%), and at visit V12: 8.9% (1.3–46.2%). In group 2, the median points of IPMDS total at V0 were: 9.65% (3.2–33.2%), and at V12: 20.45 (2.9–47.7%) (Figure 2). The difference in IPMDS points in group 1 vs. group 2 was statistically significant (*p* = 0.0048) (Figure 3 and Figure 4).

In group 1, none of the patients with diagnosed epilepsy showed increased epileptic seizure, and two patients who had seizures before inclusion in the study showed a reduced number of seizures. This group, as compared to group 2, showed improvement in muscle tone, reduced extra movements (ataxia, trembling, dystonia) and improvement in physical exercise tolerance, but the difference was not statistically significant (*p* < 0.1). Table 3 specifies clinical symptom changes in groups 1 and 2 in the follow-up period.

### 3.4. Analysis of Mitochondrial Disease Biomarker Concentration in Particular Groups

In the study population, the following MD biomarkers were measured in serum: FGF21, LA, PA, ALA and CK. Table S2 summarises data on the median and reference range for specific biomarkers in subjects with MD, measured at V0 (group 1 and group 2) and in children without MD (group 3 and group 4). The cut-off point for FGF21 in the study population (95th percentile) was 275 pg/mL.

In the follow-up period, the biochemical markers assessed in MD patients—FGF21, LA, PA, ALA, CK—did not significantly change in any group—Table S3.

A correlation between the MD stage assessed by IPMDS and the concentration of particular biomarkers of mitochondrial disease: FGF21, LA, PA, ALA and CK, was also verified. No significant correlation with the IPMDS total was shown for any of these parameters (Figure S1).

## 4. Discussion

Studies on animals and cell lines with impaired respiratory chains demonstrated that KD improves cell energy profile and increases ATP production in the mitochondrial respiratory chain [14,28]. It also leads to the stimulation of mitochondrial biogenesis in skeletal muscles and adipose tissue [29,30], prevents the formation of abnormal mitochondria, improves the structure of respiratory chain complexes (especially complex I) and increases the production of mitochondrial proteins [21,31]. It was also shown that KD could reduce the amount of COX-negative fibres (a marker of mitochondrial damage) in skeletal muscle biopsy [32,33] and reduce mtDNA heteroplasmy [31,32]. Ketone bodies improve mitochondrial dynamics in the cell—e.g., they improve mitochondrial elongation and activate mitophagy (mitochondrial autophagy), thus contributing to the removal of defective mitochondria [34]. An additional benefit, especially in MD patients, is the fact that ketone bodies are a source of energy and compensate for cell energy deficits [14,21,31,32].

KD efficacy in patients with MD has yet to be definitely confirmed, so far [35]. The first case reports of effective treatment of MD patients with KD referred to patients with pyruvate dehydrogenase deficiency (PDHD) [36]. Currently, there are numerous case reports of patients with PDHD, including one prospective study [37], which reports improvement with regard to reduced epileptic seizures, ataxia, improved gross motor skills and cognitive function. Many patients also revealed a reduced level of biochemical markers: lactic acid and/or pyruvic acid [37,38]. In isolated cases, regression of radiological changes was even demonstrated—normalisation of signal in basal ganglia on CNS MRI [39]. In the analysed study, all patients with PDHD benefited from the treatment, and none required withdrawal of KD.

Most publications assessing the efficacy of KD in patients with MD point to an improvement in the reduction of epileptic seizures. In one of the first studies, Lee Y.M. et al. showed remission of epileptic seizures in 50% of patients (in 12 of 24) treated with KD and a significant reduction in the number of seizures in another 25%, but the patients did not have genetic confirmation of MD; patients with epilepsy and reduced activity of the respiratory chain on skeletal muscle biopsy were qualified for the study [40]. In another study, Na J.H. et al. observed not only better control of epileptic seizures (seizure reduction > 50% in 8/20 patients after a year and in 7/20 patients after two years of KD) but also improved cognitive function in all patients treated with KD [41]. A few authors report remission of the status epilepticus after the introduction of KD. Still, in some patients, this effect was only temporary, and in the case of Alpers syndrome (pathogenic variant in *POLG*), KD did not improve prognosis as relates to survival [42,43].

So far, only one study has been published with a prospective assessment of KD in patients with MD, showing KD efficacy in reducing epileptic seizures in those patients, but the follow-up period was only three months, and 1/3 of the patients (10 of 33) did not complete the study [44]. In the presented study, all MD patients with active seizures treated with KD (group 1) showed significant improvement in seizure control. It was also possible to withdraw or reduce the number of antiepileptic drugs in some patients. These data are in contrast with group 2, patients with MD non-treated with KD, where the seizures did not regress in any patient (despite pharmacological treatment), and the number of seizures was even higher in two patients.

Another entity belonging to mitochondrial diseases for which efficient treatment with a ketogenic diet was shown is leukoencephalopathy conditioned by *SLC25A12* molecular variants. This gene encodes a protein which, together with other proteins (AGC1/2, OGC, GOT1/2, MDH1/2), is responsible for the structure of mitochondrial malate-aspartate shuttle (MAS). Out of 13 subjects with MAS deficiency treated with KD, clinical improvement was observed in 11 subjects, primarily in the form of a reduction in the number of epileptic seizures (reduction >50% was observed in 77% of subjects, whereas in 5/13 subjects, the seizures regressed completely). There was also improvement in muscle tone and motor development, as well as better communication and social interaction [45]. In the study group, there was one patient with MAS deficiency. She benefited greatly from the treatment with KD, i.e., significant improvement in muscle tone and motor abilities. She could also withdraw antiepileptic drugs, and an MRI (after nine months of treatment) showed improvement in the white matter myelination.

In the presented study, comparing the severity of clinical symptoms in MD patients treated with KD to those receiving standard care showed an improvement in their clinical condition assessed with IPMDS. MDs are, by definition, progressive diseases. Therefore, in the follow-up of patients with MD, one should expect an increase in the IPMDS score, as was observed in patients of group 2: 70% showed worsening clinical symptoms, and only one patient showed improvement. On the other hand, in the group of MD patients treated with KD, as many as 81% noticed improvement, and the others showed a stable condition. The improvement was manifested by, e.g., reduced number of epileptic seizures, remission or reduction in trembling, ataxia or other extra movements, and the patients or their guardians reported better functioning of the patient and improved cognitive function. Among patients treated with KD, only one person did not gain any benefits. It was a patient with mitochondrial myopathy (mtDNA deletion), but she did not show signs of rhabdomyolysis (reported in previously described adults [33]). KD was discontinued in this patient also due to signs of liver steatosis on ultrasonography, without biochemical signs of liver damage. The changes shown on ultrasound regressed after three months from KD withdrawal.

In the study group, adverse events associated with KD occurred at a comparable frequency in both MD and non-MD patients. In both groups, the most common finding was metabolic acidosis and hyperuricaemia. Most adverse reactions were mild or required a pharmacological or dietary intervention. However, only in the group of subjects with MD, it was necessary to discontinue KD due to adverse effects: acute pancreatitis, signs of hepatic steatosis on abdominal ultrasound and persisting vomiting. The complaints were remitted to all the patients after the withdrawal of KD. None of the patients revealed symptomatic hypoglycaemia, dehydration or weight loss, and frequently reported complaints during treatment with KD. Also, none of the patients were diagnosed with long QT syndrome or cardiomyopathy in the follow-up period.

## 5. Conclusions

KD is safe for patients with MD, and there are no more adverse events in the group of patients with MD than in patients treated with KD for a different reason. A ketogenic diet may be considered a therapeutic option for subjects with MD, especially those with epileptic seizures. It was shown that such treatment may improve the clinical condition of patients with MD, enabling control of epileptic seizures and slowing down MD progression. FGF21 and other MD biomarkers do not correlate with MD severity and stage, so they are not helpful for monitoring the course of treatment or assessing the stage of MD.

## Figures and Tables

**Table 1:** Data of patients in particular groups.

	Group 1 (MD + KD)	Group 2 (MD without KD)	Group 3 (KD without MD)	Group 4 (without MD, without KD)	All Groups
Number of people recruited	14	21	11	17	63
Number of people who did not complete the study	3	11	1	6	21
Number of people who completed the study	11	10	10	11	42
Median age at study entry (months); (range)	60 (10–181)	35 (2–175)	69 (18–202)	31 (8–78)	51 (2–202)
Median follow-up (months); (range)	12 (5–15)	12 (9–16)	12 (3–13)	12 (7–16)	12 (3–16)
Number of boys (%)	7 (64%)	6 (60%)	7 (70%)	6 (55%)	26 (62%)

**Table 2:** Positive effects of the ketogenic diet in the study groups.

Positive Effects of the KD	No. of Patients in Whom Improvement Was Observed/No. of Patients with a Problem (%)		All *n* = 21
	Group 1 MD + KD *n* = 11	Group 3 KD without MD *n* = 10	
Seizure reduction (total)	2/2 (100%)	6/8 (75%)	8/10 (80%)
100% reduction in seizures	1/2 (50%)	1/8 (12.5%)	2/10 (20%)
Reduction in seizures by >90%	0	0	0
Seizure reduction by 50–90%	1/2 (50%)	4/8 (50%)	5/10 (50%)
<50% reduction in seizures	0	1/8 (12.5%)	1/10 (10%)
Reduction in the number of antiepileptic drugs administered	2/5 (40%)	0/8	2/13 (15.4%)
Improvement of muscle tone	8/9 (88.8%) *	1/7 (14.3%)	9/16 (56.2%)
Reduction in extra movements (tremor, ataxia, dystonia)	5/6 (83.3%)	3/6 (50%)	8/12 (66.7%)

* Statistically significant difference *p* < 0.05 (the chi-square test of independence with Yates correction). KD—ketogenic diet, MD—mitochondrial disease, No—number.

**Table 3:** Comparison of clinical symptoms in patients with MD treated with KD (group 1) and not treated with KD (group 2).

Clinical Symptoms	No. of Patients in Whom Symptoms Was Observed/No. of All Patients (%)		All *n* = 21
	Group 1 MD + KD *n* = 11	Group 2 MD without KD *n* = 10	
Total IPMDS score reduction (clinical improvement)	9/11 (81.8%) *	2/10 (20%)	11/21 (52.4%)
Reducing the number of seizures	2/2 (100%)	0/3	2/5 (40%)
Increased number of seizures	0/5	2/5	2/10 (20%)
Reduction in the number of antiepileptic drugs	2/5 (40%)	0/5	2/10 (20%)
Improvement of muscle tone	8/9 (88.8%) **	2/6 (33.3%)	10/15 (66.6%)
Reduction in movement disorders (tremor, ataxia, dystonia)	5/6 (83.3%)	2/7 (28.6%)	7/13 (53.8%)
Improved exercise tolerance	8/11 (72.7%) **	1/7 (14.3%)	9/18 (50%)
Decrease in rate of weight gain (by 2 percentiles or <3c)	0/11	4/10 (40%)	4/21 (19%)
Decrease in height gain rate (by 2 percentile channels or <3c)	2/11 (18.2%)	0/10	2/21 (9.5%)

* Statistically significant difference *p* < 0.05, ** *p* < 0.1 (the chi-square test of independence with Yates correction). KD—ketogenic diet, MD—mitochondrial disease.

## Data Availability

All data generated or analysed during this study are included in this article and its Supplementary Material Files. Further enquiries can be directed to the corresponding author.

## Supplementary Materials

The following supporting information can be downloaded at: https://www.mdpi.com/article/10.3390/nu16060812/s1. Figure S1: Correlation between individual biomarkers of mitochondrial disease and patients’ age and disease severity (IPMDS), (Spearman’s correlation test). Table S1: Patient data in the study groups. Table S2: Biochemical parameters in patients with mitochondrial disease (group 1 + 2) and patients without disease (group 3 + 4). Table S3: Biochemical parameters in patients with mitochondrial disease at visit V0 and V12.

## Abbreviations

Ala	alanine
BHB	β-hydroxybutyric acid
CMHI-	the Children’s Memorial Health Institute
CNS	central nervous system
FGF21	fibroblast growth factor 21
IPMDS	International Pediatric Mitochondrial Disease Scale
MAD	modified Atkins diet
OXPHOS	oxidative phosphorylation process
PDHD	pyruvic acid dehydrogenase deficiency
