# Supplementary material for: Efficacy and Safety of Ketogenic Diet Treatment in Pediatric Patients with Mitochondrial Disease
Source: Nutrients. 2024 Mar 13;16(6):812. doi: 10.3390/nu16060812 (PMC10975652; doi:10.3390/nu16060812)
Supplement: Supplementary file 1 [file nutrients-16-00812-s001.zip › nutrients-2839702-supplementary.pdf]

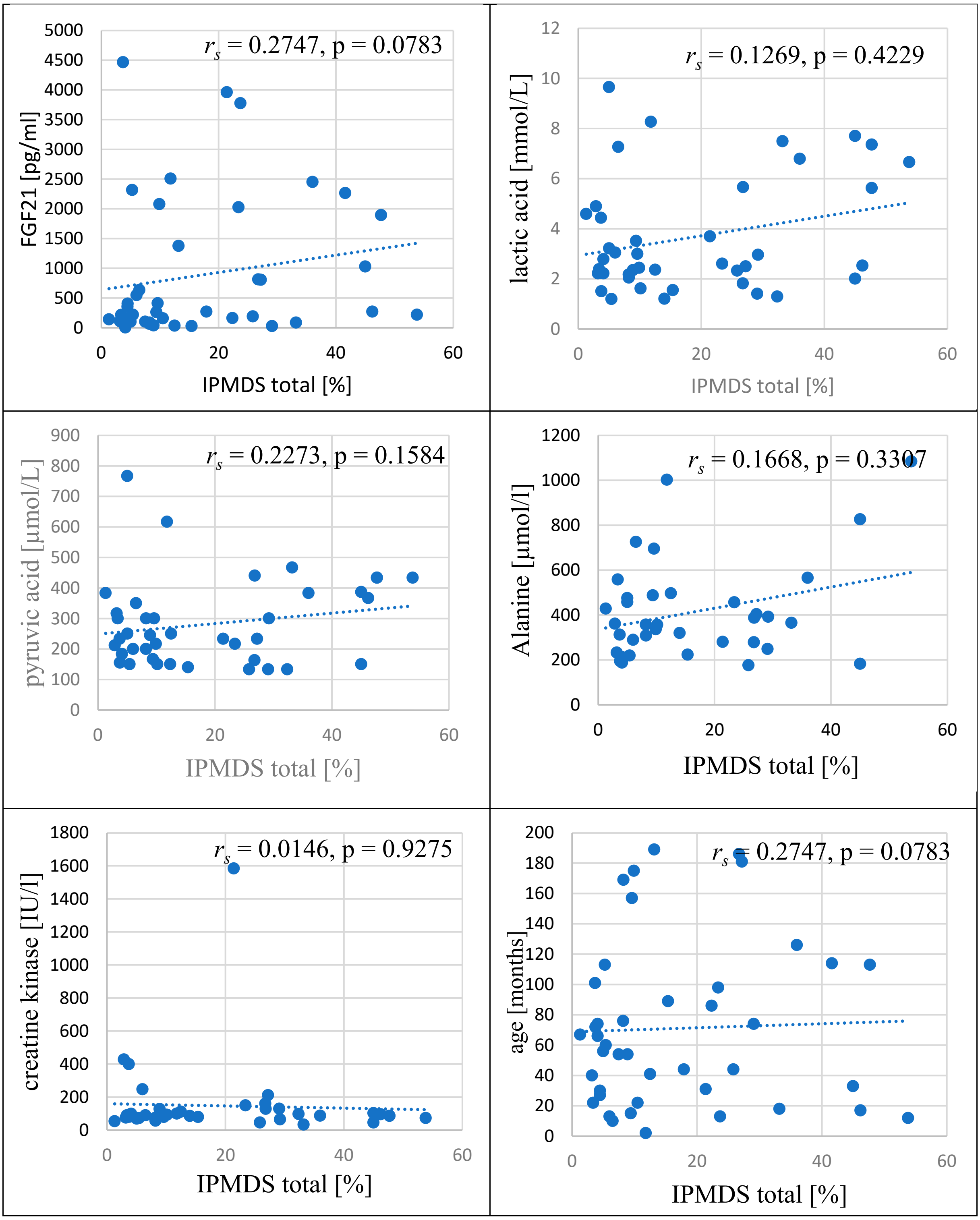

Figure S1. Correlation between individual biomarkers of mitochondrial disease and patients' age and disease severity (IPMDS)

Table S1. Patient data in the study groups

Group	No	Age at study entry (months)	Gender	Follow-up time (months)	Genetic diagnosis	Predominant clinical symptoms at study entry
Group 1: MD + KD	1.1	113	M	13	*MT-TK*	Myopathy, myoclonus, ataxia, hearing loss, short stature, PEG
	1.2	10	M	12	*PDHA1*	Delayed psychomotor development, short stature
	1.3	41	M	13	*PDHA1*	Delayed psychomotor development, ataxia, tremor, short stature
	1.4	157	M	12	*MTND5*	Intellectual disability (mild), optic nerve atrophy, epilepsy, precocious puberty, hypertrophic cardiomyopathy
	1.5	74	M	15	*PDHA1*	Tetraparesis, ataxia, tremor, peripheral neuropathy
	1.6	33	F	11	*SLC25A12*	Developmental delay, tetraparesis, hypotonia, epilepsy, dysphagia, PEG
	1.7	181	F	5	mtDNA del	Myopathy, ptosis, ataxia, tremor, hearing loss, weight deficiency
	1.8	56	F	11	*PDHA1*	Tremor, hypotonia, exercise intolerance, epilepsy, short stature
	1.9	12	M	5	*PDHA1*	Developmental delay, tetraparesis, hypotonia, refractory epilepsy, microcephaly, short stature, PEG
	1.10	60	F	12	*PDHA1*	Myalgia, exercise intolerance, short stature
	1.11	66	M	8	*BCS1L*	Exercise intolerance, muscle weakness, hearing loss, pili torti
Group 2: MD without KD	2.1	98	M	16	*MT-TK*	Myopathy, myoclonus, ataxia, hearing loss, short stature
	2.2	2	M	11	*FBXL4*	Lack of progress in psychomotor development, increased muscle tone, epilepsy, cardiomyopathy, fed by nasogastric tube
	2.3	101	F	12	*MT-TL1*	Myopathy, reduced muscle tone, short stature

	2.4	40	F	14	*PDHA1*	Tremor, ataxia, hypotone, short stature
	2.5	15	M	12	*MT-ATP6*	Delayed psychomotor development, hypotonia, cardiomyopathy, weight deficiency
	2.6	175	F	14	mtDNA del	Myopathy, intention tremor, ptosis, sensorineural hearing loss, diabetes
	2.7	13	M	9	*NUBPL*	Trochlear nerve palsy
	2.8	18	M	12	nd	Delayed psychomotor development, reduced muscle tone, epilepsy, microcephaly, short stature
	2.9	31	F	13	*COQ8A*	Delayed speech development, developmental regression, ataxia
	2.10	76	M	10	*POLG*	Epilepsy, intention tremor, post-acute liver failure
Group 3 KD without MDDK bez CM	3.1	79	M	13	*SLC2A1*	Delayed psychomotor development, clumsiness, intention tremor
	3.2	55	F	12	*ASLD*	Developmental delay, spastic tetraparesis, epileptic encephalopathy, microcephaly, PEG
	3.3	107	M	6	*MOCS1*	Spastic tetraparesis, epileptic encephalopathy
	3.4	19	M	3	*GLUL*	Delayed psychomotor development, epileptic encephalopathy
	3.5	20	M	12	nd	Delayed psychomotor development, epilepsy, microcephaly, CNS defect, ocular hypoplasia
	3.6	202	M	13	*SLC2A1*	Intellectual disability, dystonia
	3.7	77	M	12	nd	Delayed psychomotor development, spastic tetraparesis, involuntary movements, epilepsy
	3.8	62	F	13	nd	Visual impairment, hearing loss, epilepsy
	3.9	18	F	3	nd	Delayed psychomotor development, spastic

						tetraparesis, epileptic encephalopathy
	3.10	162	M	12	nd	Intellectual disability, epilepsy
Group 4 without MD and without KD	4.1	39	F	13	*RANSET2*	Spastic tetraparesis, strabismus, leukoencephalopathy
	4.2	11	M	7	nd	Delayed psychomotor development
	4.3	72	F	12	nd	Healthy, no symptoms
	4.4	11	F	16	*ADAR*	Spastic tetraparesis, encephalopathy
	4.5	11	M	10	nd	Delayed psychomotor development, epilepsy, microcephaly, CNS defect
	4.6	8	M	16	nd	Delayed psychomotor development, West syndrome
	4.7	31	M	9	nd	Delayed psychomotor development
	4.8	78	M	12	*ABCD1*	Adrenal insufficiency
	4.9	72	M	15	*OTC*	Delayed psychomotor development, epilepsy
	4.10	46	F	8	nd	Delayed psychomotor development
	4.11	31	F	15	nd	Delayed psychomotor development, ataxia

F – female KD – ketogenic diet, MD - mitochondrial disease, M – male, nd – no date, PEG – percutaneous feeding gastrostomy

Table S2. Biochemical parameters in patients with mitochondrial disease (group 1 + 2) and in patients without disease (group 3 + 4)

Biochemical parameters (normal range)	Group 1 + 2 Median (range)	Group 3 + 4 Median (range)
FGF21 [pg/mL] (normal range < 275)	412.6 (4.1 – 4466.9)	52.9 (6.1 – 294.4)
Lactic acid [mmol/L] (normal range: 0.5 – 2.2)	3.0 (1.2 – 9.6)	1.7 (0.9 – 6.0)
Pyruvic acid [µmol/L] (normal range: 50 – 200)	233.4 (133.4 – 766.9)	166.7 (66.7 – 316.7)
Alanine [µmol/L] (normal range: 144 – 418)	361 (182 – 1084)	241 (173 – 297)
Creatine kinase [IU/l] (normal range: 0 – 154)	93 (34 – 1584)	107 (38 – 972)

Table S3. Biochemical parameters in patients with mitochondrial disease at visit V0 and V12

Biochemical parameters (normal range)	Group 1 (MD + KD) Median (range) V0	Group 1 (MD + KD) Median (range)V12	Group 2 (MD without KD) Median (range)V0	Group 2 (MD without KD) Median (range)V12
FGF21 [pg/ml] (normal range < 275)	220.5 (4.1 – 1895.9)	141.2 (29.2 – 2453.0)	1288.6 (65.5 – 4466.9)	381,.3 (102.3 – 3776.0)
Lactic acid [mmol/L] (normal range: 0.5 – 2.2)	2.8 (1.2 – 9.6)	2.3 (1.5 – 6.8)	3.3 (2.2 – 8.2)	3.1 (1.2 – 7.2)
Pyruvic acid [µmol/L] (normal range: 50 – 200)	266.7 (133 – 767)	250.0 (133 – 383)	216.7 (164 – 617)	250.0 (133 – 433)
Alanine [µmol/l] (normal range: 144 – 418)	439 (182 – 1084)	278 (175 – 565)	346 (232 – 1002)	374 (319 – 826)
Creatine kinase [IU/l] (normal range: 0 – 154)	87 (46 – 212)	85 (46 – 161)	96 (34 – 1584)	90 (65 – 428)
